# Two Theorems and Important Insight on How the Preferred Mechanism of Free Radical Scavenging Cannot Be Settled. Comment on Pandithavidana, D.R.; Jayawardana, S.B. Comparative Study of Antioxidant Potential of Selected Dietary Vitamins; Computational Insights. *Molecules* 2019, *24*, 1646

**DOI:** 10.3390/molecules27228092

**Published:** 2022-11-21

**Authors:** Ioan Bâldea

**Affiliations:** Theoretical Chemistry, Heidelberg University, Im Neuenheimer Feld 229, D-69120 Heidelberg, Germany; ioan.baldea@pci.uni-heidelberg.de

**Keywords:** radical scavenging activity, antioxidant mechanisms, HAT, SPLET, SET-PT, BDE, IP, PDE, PA, ETE, thermochemistry, quantum chemistry

## Abstract

Totally ignoring that the five enthalpies of reaction—bond dissociation enthalpy (BDE), adiabatic ionization potential (IP), proton dissociation enthalpy (PDE), proton affinity (PA), and electron transfer enthalpy (ETE)—characterizing the three free radical scavenging mechanisms—direct hydrogen atom transfer (HAT), sequential electron transfer proton transfer (SET-PT), and stepwise proton loss electron transfer (SPLET)—are not independent of each other, a recent publication on the antioxidant activity of dietary vitamins compared various vitamins and “found” different quantities, which should be strictly equal by virtue of energy conservation. Aiming to clarify this point, as well as to avoid such mistakes in future studies and to unravel errors in the previous literature, in the present paper we formulate two theorems that any sound results on antioxidation should obey. The first theorem states that the sums of the enthalpies characterizing the individual steps of SET-PT and SPLET are equal: IP+PDE = PA+ETE (=H2). This is a mathematical identity emerging from the fact that both the reactants and the final products of SET-PT and SPLET are chemically identical. The second theorem, which is also a mathematical identity, states that H2 − BDE = IP${}_{H}$ > 0, where IP${}_{H}$ is the ionization potential of the H-atom in the medium (e.g., gas or solvent) considered. Due to their general character, these theorems may/should serve as necessary sanity tests for any results on antioxidant activity, whatever the method employed in their derivation. From a more general perspective, they should represent a serious word of caution regarding attempts to assign the preferred free radical scavenging pathway based merely on thermochemical descriptors.

## 1. Introduction

Oxidative stress is an extremely dangerous phenomenon caused by the rapid production of free radicals (R${}^{•}$) in the human body [1,2,3,4,5]. Owing to their unpaired electron, free radicals can seriously damage a variety of biomolecules related to a plethora of pathological processes, including, but not limited to, cardiovascular and neurodegenerative diseases [6,7,8,9,10,11,12,13,14,15,16,17]. Antioxidants (AXH) are molecules that can scavenge free radicals by H-atom donation (AXH + R${}^{•}$ → AX${}^{•}$ + RH), and can neutralize free radicals through single-step (direct H-atom transfer, HAT) or two-step mechanisms. The latter can proceed either via stepwise electron transfer–proton transfer (SET-PT) or via sequential proton loss–electron transfer (SPLET). A certain environment (solvent polarity) may selectively favor one over the other aforementioned pathways. By and large, there is a consensus that free radical scavenging by bio-relevant antioxidants (e.g., dietary vitamins and drugs against lipoprotein oxidation) proceeds as a single-step process (HAT) in non-polar solvents and as a two-step process in polar solvents. The most “popular” disputes (for vitamin E, see, e.g., [18,19,20]) on the antioxidant activity are on whether SET-PT or SPLET prevails in polar media. The most frequent challenge related to this dilemma is that differentiating between SET-PT and SPLET is a difficult issue that cannot be merely couched in terms of the antioxidant’s thermodynamical descriptors.

An adequate analysis of the preferred radical scavenging mechanism should not only consider the properties of the antioxidant but also the properties of the radical [19,21,22]. The conclusions of such an analysis can be surprising, as in the case of atorvastatin-based species [22].

In this vein, it was the recent claim made in [23] (even in the abstract) that HAT, rather than SET-PT or SPLET, is the preferred pathway along which dietary vitamins scavenge free radicals in aqueous solution—without any specific consideration of the free radicals to be neutralized—that attracted our attention. As elaborated below, this is only one of the severe flaws that plague [23]. An even more important source of errors in the reported results is the fact that [23] completely overlooked and failed to adequately account for the fact that even the thermodynamical descriptors characterizing the free radical scavenging activity of a given antioxidant are not molecular properties independent of each other. This is exactly what is stated by the two theorems presented below.

To avoid creating the impression that the present work is a “declaration of war” to the free radical/antioxidant community, we will deliberately restrict ourselves to naming a few previous works [23,24,25] and show how the two theorems, used as sanity checks, can easily unravel inadequate results of quantum chemical calculations. The fact that we are going to refer below to the works [23,24,25] (out of many others) should by no means be understood as an “attack” on these studies. It should become clear from the analysis that follows that these theorems are of great significance in diverse areas of chemistry.

## 2. Computational Details

The quantum chemical calculations carried out in conjunction with the specific molecule (vitamin B3) considered in this study used the GAUSSIAN 16 suite of programs [26], similar to those in our recent study on atorvastatin [22]. In order to make this paper self-contained, they will be briefly described again.

Geometry optimizations without constraints, frequency calculations (checking that all vibrational frequencies were real), and electronic energy calculations were carried out at the DFT level of theory using the hybrid B3LYP exchange correlation functional [27,28,29,30] and 6-311G(d,p) and 6-311++G(d,p) basis sets [31,32]. The solvent (water) was treated within the polarized continuum model (PCM) [33] using the integral equation formalism (IEF) [34]. Similar to other cases studied recently [35,36,37], spin contamination did not appear to be an issue for the unrestricted spin (UB3LYP) approaches, as witnessed by the values of the total spin values $\langle S^{2}\rangle$ before and after the annihilation of the first spin contaminant presented in the following. For reasons explained below, we also performed restricted open-shell (ROB3LYP) calculations, which are more computationally demanding for larger molecules. The small differences between the unrestricted and restricted open-shell methods revealed that dynamic electron correlations brought about by spin polarization effects are weak; still, it should be made clear that claims (often formulated in the literature on antioxidation) of chemical accuracy (∼1 kcal/mol) are totally unrealistic. Achieving chemical accuracy for bond dissociation enthalpies and proton affinity (BDE and PA, quantities entering the discussion that follows) is often illusory, even for extremely computationally demanding state-of-the-art compound model chemistries (CBS-QB3, CBS-APNO, G4, W1BD) and smaller molecular sizes [38].

All enthalpies listed below refer to the temperature T=298.15 K.

## 3. Results and Discussion

### 3.1. Enthalpies of Reaction Characterizing the Antioxidant Activity

As noted in the Introduction, an antioxidant can transfer an H-atom to a free radical in one- or two-step processes. Again, to make the paper self-contained, let us be reminded that the three antioxidative mechanisms (HAT, SET-PT, and SPLET) and the corresponding reaction enthalpies (BDE, IP and PDE, and PA and ETE, respectively) can be expressed as follows:

Direct hydrogen atom transfer (HAT) [39,40,41]

(1) $$\begin{array}{cc} AXH+R^{•}\to {AX}^{•}+RH & BDE=H\left({AX}^{•}\right)+H\left(H^{•}\right)-H\left(AXH\right). \end{array}$$

Stepwise electron transfer–proton transfer (SET-PT) [42,43]

(2a) $$\begin{array}{cc} AXH\to AXH^{•+}+e^{-} & IP=H\left(AXH^{•+}\right)+H\left(e^{-}\right)-H\left(AXH\right). \end{array}$$

(2b) $$\begin{array}{cc} AXH^{•+}\to {AX}^{•}+H^{+} & PDE=H\left({AX}^{•}\right)+H\left(H^{+}\right)-H\left(AXH^{•+}\right). \end{array}$$

Sequential proton loss–electron transfer (SPLET) [44,45]

(3a) $$\begin{array}{cc} AXH\to {AX}^{-}+H^{+} & PA=H\left({AX}^{-}\right)+H\left(H^{+}\right)-H\left(AXH\right) \end{array}$$

(3b) $$\begin{array}{cc} {AX}^{-}\to {AX}^{•}+e^{-} & ETE=H\left({AX}^{•}\right)+H\left(e^{-}\right)-H\left({AX}^{-}\right). \end{array}$$

In specific cases of interest for antioxidation, X stands for an O, N, or S atom. The above definitions should make clear that all aforementioned reaction enthalpies (in particular, IP) are adiabatic rather than vertical properties [46,47]; they should be evaluated at the global electronic energy minima of the various reaction products/reactants.

### 3.2. Theorems on Antioxidation

The first theorem stated below expresses the chemical fact that both the reactants and the final products of the two two-step antioxidative mechanisms are identical.

**Theorem** **1.** *Whatever the antioxidant and the environment, the combined enthalpies pertaining to the two-step SET-PT and SPLET mechanisms are equal:*

(4) $$\underset{SETPT}{\underset{︸}{\mathrm{IP}+\mathrm{PDE}}}=\underset{SPLET}{\underset{︸}{\mathrm{PA}+\mathrm{ETE}}}.$$

**Proof of Theorem 1.**  The theorem straightforwardly follows by adding, term by term, the left- and right-hand sides of Equations (2a), (2b), (3a) and (3b)

(5a) $$\begin{array}{ccc} IP+PDE & = & H\left(\begin{array}{c} e^{-} \end{array}\right)-H\left(\begin{array}{c} AXH \end{array}\right)+H\left(\begin{array}{c} {AX}^{•} \end{array}\right)+H\left(\begin{array}{c} H^{+} \end{array}\right) \end{array}$$

(5b) $$\begin{array}{ccc} PA+ETE & = & H\left(\begin{array}{c} H^{+} \end{array}\right)-H\left(\begin{array}{c} AXH \end{array}\right)+H\left(\begin{array}{c} {AX}^{•} \end{array}\right)+H\left(\begin{array}{c} e^{-} \end{array}\right). \end{array}$$

As is visible, the right-hand sides of Equations (5a) and (5b) are identical. □

**Corollary** **1.**
(6a) $$\begin{array}{ccc} \mathrm{IP} & < & \mathrm{PA}\Leftrightarrow \mathrm{PDE}>\mathrm{ETE} \end{array}$$

(6b) $$\begin{array}{ccc} \mathrm{IP} & > & \mathrm{PA}\Leftrightarrow \mathrm{PDE}<\mathrm{ETE} \end{array}$$

(6c) $$\begin{array}{ccc} \mathrm{IP} & < & \mathrm{ETE}\Leftrightarrow \mathrm{PDE}>\mathrm{PA} \end{array}$$

(6d) $$\begin{array}{ccc} \mathrm{IP} & > & \mathrm{ETE}\Leftrightarrow \mathrm{PDE}<\mathrm{PA}. \end{array}$$

The second theorem establishes a relationship between the enthalpies of reactions characterizing the single-step and two-step antioxidative mechanisms.

**Theorem** **2.** *The combined enthalpy of reaction pertaining to the two-step (SET-PT and SPLET) mechanisms exceeds the enthalpy of the direct H-atom transfer reaction. Whatever the antioxidant, the difference is equal to the ionization enthalpy of the H-atom ${IP}_{H}$ in the corresponding environment:*

(7) $$\mathrm{IP}+\mathrm{PDE}=\mathrm{PA}+\mathrm{ETE}=\mathrm{BDE}+{\mathrm{IP}}_{H}>\mathrm{BDE}.$$

**Proof of Theorem 2.**  The theorem straightforwardly follows by subtracting, term by term, the left- and right-hand sides of Equations (2) (or Equation (3)) and (1), and observing that the difference thus obtained represents the ionization enthalpy of the hydrogen atom:

(8) $$IP+PDE-BDE=PA+ETE-BDE=\underset{{IP}_{H}}{\underset{︸}{H\left(H^{+}\right)+H\left(e^{-}\right)-H\left(H^{•}\right)}}.$$

□

**Corollary** **2.** *Being equal to the ionization enthalpy of the H-atom (cf. Equation (8)), the difference between the enthalpies of the total two- and single-step pathways merely depends on the medium; it is the same for all antioxidant species*

(9) $$\begin{array}{ccc} \mathrm{IP}+\mathrm{PDE}-\mathrm{BDE} & = & \mathrm{PA}+\mathrm{ETE}-\mathrm{BDE}={\mathrm{IP}}_{H}>0\\ & = & same\;positive\;\underline{value}\;for\;all\;antioxidants\;in\;a\;given\;environment\;\left(solvent\right). \end{array}$$

**Remark** **1.** *Remark on Theorems 1 and 2. We referred above to enthalpies of reaction because these are usually examined in studies on antioxidants, but Gibbs free enthalpies satisfy the same equations.*

### 3.3. Implications of Theorem 1

The results presented in the literature on antioxidants violating Theorem 1 (and hence being incorrect) fall into two categories.

Studies reporting values SETPT=IP+PDE different from SPLET=PA+ETE belong to the first category. Because the main aim of this work is to draw attention to the necessary sanity tests rather than amply documenting incorrect results on antioxidative activity reported in the literature in the past, we will only give here a single but notorious example, to which, unpleasantly, we return to repeatedly below.

We named [23], which is a notorious case because, unfortunately, *all* values presented for the dietary vitamins investigated (A, B1, B3, B6, and C) fail to obey Theorem 1. This is obvious by inspecting the last column of Table 1, where the pertaining results are collected. It is pure nonsense to settle between SET-PT and SPLET by “comparing” (as in [23]) the combined enthalpies of the pertaining reactions IP+PDE and PA+ETE. Provided that they are correctly estimated, they should be strictly equal to each other, whatever the computational method utilized.

The second category comprises studies adjudicating between SET-PT and SPLET based on the reaction enthalpy of the first step. For example, SPLET is claimed to be the thermodynamically preferred pathway because PA<IP. This is incorrect because it contradicts Corollary 1. If this was the case, thermodynamically speaking, the second, rate-determining step (electron transfer, ETE) would act as bottleneck of SPLET (ETE>PDE, cf. Equation (6b)).

Equation (6) makes it clear that in all cases, it is the specific free radical and/or the reaction kinetics that settles whether SET-PL or SPLET prevails. Merely considering antioxidant’s enthalpies of reaction can never discriminate between these two pathways.

### 3.4. Implications of Theorem 2

There are at least two practical issues related to Theorem 2.

First, the theorem shows that it is incorrect to assign HAT as the preferred antioxidant pathway based on the fact that BDE is smaller than IP+PDE (or PA+ETE). Theorem 2 states that correctly estimated enthalpies should *always* satisfy this inequality. This refutes the claim made in [23] on this basis, that HAT is the thermodynamically preferred pathway for dietary vitamins to scavenge free radicals in aqueous solution.

Above, we said “correctly estimated” because, unfortunately, this is not always the case in the literature; for example, in [25], where enormous values of BDE≈400 kcal/mol, much larger than all the other reaction enthalpies, were obtained (see [22,48]). At odds with Equation (7), the largest value of IP+PDE=PA+ETE reported in the mentioned study are smaller than BDE; none exceeds ∼150 kcal/mol (cf. Table 2 of [25]).

Second, Theorem 2 emphasizes the particularly important role of the ionization enthalpy of the H-atom ${IP}_{H}$. For this reason, in Table 2 we present extensive data for the enthalpy of the H-atom. We used these data in Table 3 and Table 4 for computing ${IP}_{H}$ in gas and solvents using popular functionals (B3LYP, PBE0, M052x, and M062x) and frequently employed Pople basis sets.

Regarding the values of ${IP}_{H}$, a twofold word of caution is in order.

First, estimating ${IP}_{H}\approx -E_{HOMO}^{KS}$ as the Kohn–Sham HOMO energy with reversed sign (Koopmans’ theorem) lamentably fails in a twofold sense; see Table 5 and Table 6. Because Kohn–Sham “orbitals” are mathematical objects rather than true molecular orbitals [47,49], these estimates are inadequate. The differences between the B3LYP- and PBE0-based Koopmans’ values and the Δ-DFT-based values [50,51,52] are unacceptable even in the gas phase: ∼4–5 eV for B3LYP and PBE0 (cf. Table 5) and ∼3.5 eV for M062x and M052x (cf. Table 6). These differences grow to enormous values in polar solvents (∼12 eV, cf. Table 5 and Table 6). This dramatic deterioration of the description based on Koopmans’ theorem is fully in line with findings reported in previous studies on molecules in solvents [46,53].

Second, differences in the values of antioxidant thermodynamic descriptors in solvents as large as ∼0.5 eV (12 kcal/mol, 50 kJ/mol) reported in various publications do not necessarily reflect real physical and chemical differences. They can simply stem from the utilization of different values of the electron and/or hydrogen atom enthalpy of solvation circulated in the literature (e.g., $\Delta_{solv}H\left(\begin{array}{c} H^{+} \end{array}\right)=-1022$ kJ/mol [54] versus $\Delta_{solv}H\left(\begin{array}{c} H^{+} \end{array}\right)$=−1058.9 kJ/mol [55] for a proton in water).

According to Theorem 2, the difference between IP+PDE=PA+ETE and BDE should be equal to the ionization enthalpy of the H-atom ${IP}_{H}$ in the medium (solvent) in question. It should be, but unfortunately, data in the literature exist for which this condition is not fulfilled.

In the upper part of Table 7, we reproduce enthalpies reported at the B3LYP/6-31+G(d,p)(/IEFPCM) level of theory for the natural food colorant peonidin in gas and aqueous phases [24].

It might be questionable whether the difference between the values IP+PDE = 392.87 kcal/mol and PA+ETE=394.42 kcal/mol represents an obscure numerical artifact or a violation of Theorem 1. In fact, this should not be the case, given the fact that Theorem 1 is a mathematical identity that must be satisfied at any approximate level of theory provided that all antioxidant descriptors were correctly computed using the same method.

Anyway, letting alone this aspect, the values estimated for IP+PDE−BDE=313.95 kcal/mol and PA+ETE−BDE=315.50 kcal/mol differ too much from IP${}_{H}$=32.8 kcal/mol; they obviously do not satisfy Theorem 2.

Pleasantly, no objection can be raised against the enthalpies estimated in [24] for peonidin in the gas phase. They satisfy both Theorems 1 and 2.

Let us next refer again to notorious case of [23]. Relevant results are depicted in Table 1. The inspection of the fifth column of Table 1 reveals values that drastically differ from the ionization enthalpy of the H-atom in water (cf. Table 4). Furthermore, those values also significantly depend on the molecular species, which should not be the case if they were correct.

**Figure 1:**
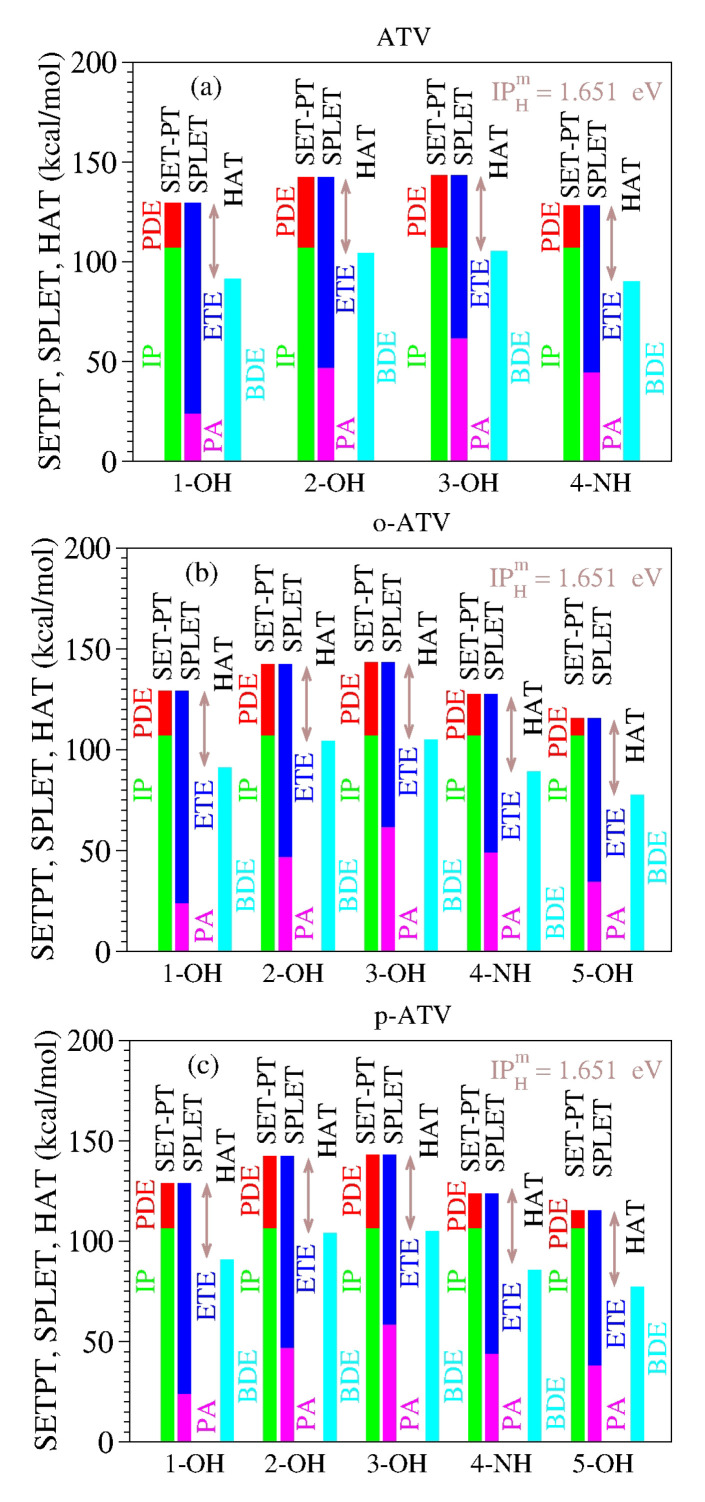
The enthalpies of reaction for (**a**) atorvastatin (ATV) and its (**b**) ortho-hydroxy (o-ATV) and (**c**) para-hydroxy (p-ATV) metabolites [22] satisfy the two theorems of antioxidation discussed in this paper. See the main text for details.

As a specific illustration of the latter aspect noted above, let us examine the results computed for atorvastatin (ATV) and its ortho- and para-hydroxy metabolites (o-ATV, p-ATV) in methanol at the B3LYP/6-31+G(d,p) level of theory [22]. As visible in Table 8 and Figure 1, for each molecular species (ATV, o-ATV, and p-ATV) and any possible H-atom donation—that is, for each of the three (ATV) or four (o-ATV, p-ATV) OH-groups and the NH-group (indicated for the molecular geometries presented in [22])—the results of the ATV-based species satisfy both theorems.

### 3.5. Detailed Analysis of a Specific Case: Vitamin B3

**Figure 2:**
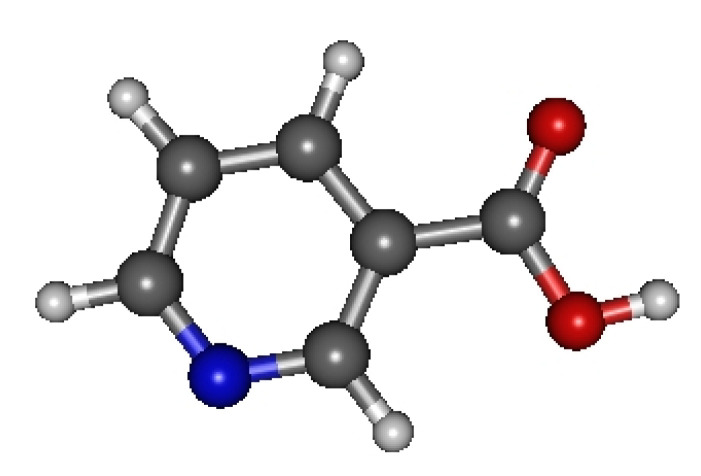
Optimized geometry of vitamin B3 (C_6_H_5_NO_2_). Figure generated using GABEDIT [56].

Let us next examine in detail the case of vitamin B3 (C_6_H_5_NO_2_, InChI=1S/C6H5NO2/ c8-6(9)5-2-1-3-7-4-5/h1-4H,(H,8,9), CAS Registry Number: 59-67-6), whose optimized geometry is depicted in Figure 2. Vitamin B3 is one of the molecular species investigated in [23]. The analysis that follows shows that, as it should be in general, the antioxidant properties of vitamin B3 also obey the two theorems presented above.

In our calculations for vitamin B3, we attempted to ensure consistency with our recent [22,48] and ongoing investigations on antioxidation, while also gaining insight into specific sources of the erroneous results reported in [23]. For the first reason, we used the B3LYP exchange-correlation functional, for which results for vitamin B3 are also presented in [23] (cf. Table 1 of the supplementary material of [23]).

Ambiguities arose regarding both the basis set—6-311++G(d,p) according to Section 3 but 6-311G**(≡6-311G(d,p)) according to Table 1 in the supplementary material of [23]— and the—unrestricted (UB3LYP) or spin-restricted open-shell (ROB3LYP)—method employed for radicals in [23]. (Unfortunately, our inquiry on these aspects to the authors of [23] continues to be pending.)

**Figure 3:**
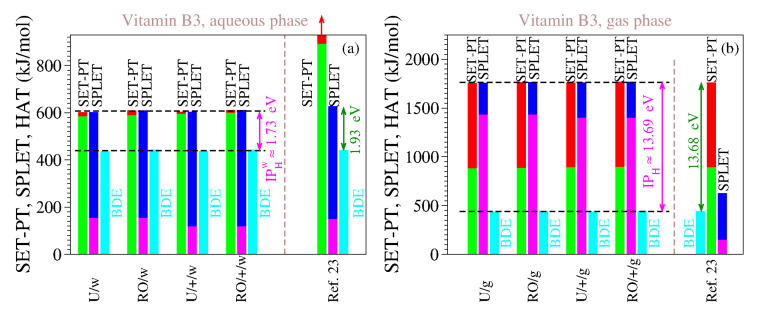
The same as Figure 1 but for vitamin B3 in the (**a**) aqueous phase and (**b**) gaseous phase. The numerical values underlying this figure are indicated in Table 9 and Table 10.

For the reasons delineated above, we computed the antioxidant descriptors of vitamin B3 using both methods (UB3LYP and ROB3LYP) and both basis sets (6-311++G(d,p) and 6-311G(d,p)). The results are presented in Table 9 and Table 10, and Figure 3. The inspection of Table 9 and Table 10 and Figure 3 reveals the clear differences between our estimates and those of [23], which are also shown. As is visible, both for the gaseous phase and for the aqueous phase our values do satisfy Theorems 1 and 2.

We also performed computations for the gas phase because the enormous values of IP and PDE of [23] (cf. Table 9) made us suspicious that, contrary to what [23] asserted, they were not computed for the aqueous phase but rather for the gaseous phase. It is, so far, an open question whether our assumption holds true or not, but the fact is that the IP and PDE of [23] are completely different from our IP and PDE in water, while being considerably closer to our estimates for the gas phase. This also holds true for all the other vitamins investigated in [23]; see Table 1.

Parenthetically (because this is not the primary aim of the present work), we can still mention a couple more incorrect statements in [23]. For example, the claim on page 5 of [23], that the first step of the SET-PT mechanism (i.e., Equation (2a)) is less energetically costly than the second SET-PT step (i.e., Equation (2b)), and another claim on page 6 that the production of the alkoxide anion in the first SPLET mechanism (i.e., Equation (3a)) requires more energy than that for the electron transfer from the alkoxide anion to the free radical (i.e., Equation (3b)). In reality, the inspection of the thermochemical data for the aqueous phase (which [23] considered) in Table 9 illustrates that just the opposite holds true (namely, IP>PDE and PA<ETE).

### 3.6. Remark on the Dominant Antioxidant Mechanism

Theorem 1 makes it clear why the discussion on the competition between SET-PT and SPLET is incorrect in [23]. There, the former mechanism was ruled out due to the large values of IP (in fact, incorrectly computed, see Table 9), much larger than PA. In general, if this were the case, Theorem 1 would necessarily imply that PDE≪ETE, which means that the second step of SPLET would then act as a bottleneck for the SPLET pathway. To reiterate, discussing the competition SET-PT versus SPLET merely based on the enthalpies of reaction is impossible, simply because the combined enthalpies of the two pathways (SET-PT and SPLET) are strictly equal in all cases.

The claim made in [23] (even in the abstract) that HAT prevails is not substantiated by the values (even if they were correct) presented in that work. From the fact that BDE<PA+ETE, one can/should by no means conclude that HAT prevails over SPLET. This inequality holds in *all* cases (cf. Equation (9)).

On the other hand, as emphasized recently [22], a “small” value of PA (implicitly meaning a PA smaller than BDE) does not make SPLET a thermodynamically allowed pathway, per se. The relationship between BDE and PA plays absolutely no role in the the first step of SPLET (proton loss). What matters in the first SPLET reaction is that the antioxidant’s PA be smaller than the PDE value of the neutralized free radical; see the discussion in [22]. Again, whether SPLET is allowed or not cannot be settled merely in terms of the antioxidant’s properties.

## 4. Conclusions

By presenting extensive data for the H-atom ionization potential obtained with frequently utilized functionals and basis sets (Table 3 and Table 4), we aimed to provide the reader with a toolkit useful for performing expedient sanity checks of published (or own) results on antioxidant thermochemistry relying on Theorems 1 and 2.

It was not our main purpose to provide an extensive documentation of errors in previous studies on free radical scavenging. By intentionally restricting ourselves to mention a very limited number of recent works [23,24,25] plagued by severe flaws, we primarily aimed to draw attention to the fact that such errors exist and, hence, that an overall word of caution is strongly recommended. Assisting the interested reader in quickly checking the (in)correctness of results on antioxidation reported in the previous literature on free radical scavenging is unfortunately a nontrivial utility of these theorems. We say “unfortunately” because a closer look at many publications may still be a source of numerous unpleasant surprises.

From a more general perspective, we believe that the two theorems formulated in this paper are important for several reasons. The most straightforward consequence is the clear demonstration that discriminating between SET-PT and SPLET—a “favorite” dilemma in the field of free radical scavenging—is impossible merely based on the reaction enthalpies characterizing the antioxidant. To this aim, letting alone aspects of kinetics—which are of potential, paramount importance—additional information on the thermochemical properties of the free radicals envisaged is indispensable.

## Figures and Tables

**Table 1:** The enthalpies of reaction (in kJ/mol) for dietary vitamins in aqueous solution taken from Table 1 of [23] violate Theorem 1; the values in the fourth column should be zero (cf. Equation (4)) but they are not. They also violate Theorem 2; all values of the fifth column should be equal to the H-atom ionization enthalpy in water (sixth column) but they are closer to that in the gas phase (seventh column). All quantities shown here are averages of the values computed using the Minnesota functionals M052x and M062x.

Molecule	IP + PDE	PA + ETE	IP + PDE − (PA + ETE)	IP + PDE − BDE	${\mathrm{IP}}_{H}^{\;\mathrm{water}}$	${\mathrm{IP}}_{H}^{\;\mathrm{gas}}$
Vitamin A	1742.0±2.0	532.0±1.0	1210.0≠0	1386.0	155.6	1312.6
Vitamin B1	1750.5±0.5	611.5±1.5	1139.0≠0	1315.5	155.6	1312.6
Vitamin B3	1790.5±1.5	658.0±2.0	1132.5≠0	1309.0	155.6	1312.6
Vitamin B6	1655.0±1.0	537.0±0.0	1118.0≠0	1294.5	155.6	1312.6
Vitamin C	1611.5±1.5	495.0±2.0	1116.5≠0	1293.0	155.6	1312.6

**Table 2:** Enthalpies of the H-atom at T=298.15 K obtained by adding the thermal correction of 0.002361 hartree (common for all compound model chemistries from GAUSSIAN 16) to the electronic energies, computed as indicated below. As visible here, notwithstanding the different number of basis functions, the results using the basis sets 6-311+G(d,p) and 6-311G(d,p), and 6-31+G(d,p) and 6-31G(d,p) are identical. Likewise, including more polarization functions, G(d,p) → G(2d,2p) or 6-311++(3df,3pd), has no impact on the values presented here.

Functional	Basis Set	*H*(H) (Hartree)
B3LYP	6-311++G(3df,3pd)	−0.499896
	6-311++G(2d,2p)	
	6-311++G(d,p)	
	6-311+G(3df,3pd)	−0.499795
	6-311+G(2d,2p)	
	6-311+G(d,p)	
	6-311G(3df,3pd)	
	6-311G(2d,2p)	
	6-311G(d,p)	
	6-31++G(d,p)	−0.499305
	6-31+G(d,p)	−0.497912
	6-31G(d,p)	
PBE0	6-311++G(3df,3pd)	−0.498787
	6-311++G(2d,2p)	
	6-311++G(d,p)	
	6-311+G(3df,3pd)	−0.498677
	6-311+G(2d,2p)	
	6-311+G(d,p)	
	6-311G(3df,3pd)	
	6-311G(2d,2p)	
	6-311G(d,p)	
	6-31++G(d,p)	−0.498050
	6-31G+(d,p)	−0.496748
	6-31G(d,p)	
M062x	6-311++G(3df,3pd)	−0.495834
	6-311++G(2d,2p)	
	6-311++G(d,p)	
	6-311+G(3df,pd)	−0.495773
	6-311+G(2d,2p)	
	6-311+G(d,p)	
	6-311G(3df,3pd)	
	6-311G(2d,2p)	
	6-311G(d,p)	
	6-31++G(d,p)	−0.495941
	6-31+G(d,p)	−0.494305
	6-31G(d,p)	
M052x	6-311++G(3df,3pd)	−0.496932
	6-311++G(2d,2p)	
	6-311++G(d,p)	
	6-311+G(3df,3pd)	−0.496847
	6-311+G(2d,2p)	
	6-311+G(d,p)	
	6-311G(3df,3pd)	
	6-311G(2d,2p)	
	6-311G(d,p)	
	6-31++G(d,p)	−0.497087
	6-31+G(d,p)	−0.495545
	6-31G(d,p)	

**Table 3:** Ionization enthalpies (IP${}_{H}$) of the H-atom in common environments. As already noted in the caption of Table 2, the results using the basis sets 6-311+G(d,p) and 6-311G(d,p), and 6-31+G(d,p) and 6-31G(d,p) are identical. Likewise, including more polarization functions, G(d,p) → G(2d,2p) or 6-311++(3df,3pd), has no impact on the values presented here.

Medium	Method	kcal/mol	kJ/mol	eV
Gas	B3LYP/6-311++G(d,p)	315.9	1321.8	13.699
$H(H^{+})=6.140$ kJ/mol ${}^{a}$	B3LYP/6-311+G(d,p)	315.8	1321.5	13.696
$H(e^{-})=3.135$ kJ/mol ${}^{a}$	B3LYP/6-31++G(d,p)	315.5	1320.2	13.683
	B3LYP/6-31+G(d,p)	314.7	1316.5	13.645
	PBE0/6-311++G(d,p)	315.2	1318.8	13.669
	PBE0/6-311+G(d,p)	315.8	1321.5	13.696
	PBE0/6-31++G(d,p)	314.7	1316.9	13.649
	PBE0/6-31+G(d,p)	313.9	1313.5	13.613
	M062x/6-311++G(d,p)	313.4	1311.1	13.589
	M062x/6-311+G(d,p)	313.3	1310.9	13.587
	M062x/6-31++G(d,p)	313.4	1311.4	13.591
	M062x/6-31+G(d,p)	312.4	1307.1	13.547
	M052x/6-311++G(d,p)	314.0	1314.0	13.618
	M052x/6-311+G(d,p)	314.0	1313.8	13.616
	M052x/6-31++G(d,p)	314.1	1314.4	13.623
	M052x/6-31+G(d,p)	313.2	1310.3	13.581
Benzene	B3LYP/6-311++G(d,p)	93.9	393.0	4.073
$\Delta H_{sol}\left(H^{+}\right)=-904.9$ kJ/mol ${}^{b}$	B3LYP/6-311+G(d,p)	93.9	392.7	4.070
$\Delta H_{sol}\left(e^{-}\right)=-17.5$ kJ/mol ${}^{b}$	B3LYP/6-31++G(d,p)	93.5	391.4	4.057
$\Delta H_{sol}\left(H\right)=6.4$ kJ/mol ${}^{a}$	B3LYP/6-31+G(d,p)	92.7	387.7	4.019
	PBE0/6-311++G(d,p)	93.2	390.0	4.042
	PBE0/6-311+G(d,p)	93.9	392.7	4.070
	PBE0/6-31++G(d,p)	92.8	388.1	4.022
	PBE0/6-31+G(d,p)	91.9	384.7	3.987
	M062x/6-311++G(d,p)	91.4	382.3	3.962
	M062x/6-311+G(d,p)	91.3	382.1	3.961
	M062x/6-31++G(d,p)	91.4	382.6	3.965
	M062x/6-31+G(d,p)	90.4	378.3	3.921
	M052x/6-311++G(d,p)	92.1	385.2	3.992
	M052x/6-311+G(d,p)	92.0	384.9	3.990
	M052x/6-31++G(d,p)	92.2	385.6	3.996
	M052x/6-31+G(d,p)	91.2	381.5	3.954
Toluene	B3LYP/6-311++G(d,p)	84.8	354.9	3.678
$\Delta H_{sol}\left(H^{+}\right)=-939.1$ kJ/mol ${}^{b}$	B3LYP/6-311+G(d,p)	84.7	354.6	3.675
$\Delta H_{sol}\left(e^{-}\right)=-22.7$ kJ/mol ${}^{b}$	B3LYP/6-31++G(d,p)	84.4	353.3	3.662
$\Delta H_{sol}\left(H\right)=5.1$ kJ/mol ${}^{a}$	B3LYP/6-31+G(d,p)	83.6	349.6	3.624
	PBE0/6-311++G(d,p)	84.1	351.9	3.648
	PBE0/6-311+G(d,p)	84.7	354.6	3.675
	PBE0/6-31++G(d,p)	83.7	350.0	3.628
	PBE0/6-31+G(d,p)	82.8	346.6	3.592
	M062x/6-311++G(d,p)	82.3	344.2	3.567
	M062x/6-311+G(d,p)	82.2	344.0	3.566
	M062x/6-31++G(d,p)	82.3	344.5	3.570
	M062x/6-31+G(d,p)	81.3	340.2	3.526
	M052x/6-311++G(d,p)	83.0	347.1	3.597
	M052x/6-311+G(d,p)	82.9	346.8	3.595
	M052x/6-31++G(d,p)	83.0	347.5	3.601
	M052x/6-31+G(d,p)	82.1	343.4	3.559

^a^ Cf. Table 3, [54]. ^b^ Table 5, [55].

**Table 4:** Ionization enthalpies (IP${}_{H}$) of the H-atom in common solvents. As already noted in the caption of Table 2, the results using the basis sets 6-311+G(d,p) and 6-311G(d,p), and 6-31+G(d,p) and 6-31G(d,p) are identical. Likewise, including more polarization functions, G(d,p) → G(2d,2p) or 6-311++(3df,3pd), has no impact on the values presented here.

Medium	Method	kcal/mol	kJ/mol	eV
Ethanol	B3LYP/6-311++G(d,p)	41.0	171.5	1.777
$\Delta H_{sol}\left(H^{+}\right)=-1071.3$ kJ/mol ${}^{b}$	B3LYP/6-311+G(d,p)	40.9	171.2	1.774
$\Delta H_{sol}\left(e^{-}\right)=-75.3$ kJ/mol ${}^{b}$	B3LYP/6-31++G(d,p)	40.6	169.9	1.761
$\Delta H_{sol}\left(H\right)=3.7$ kJ/mol ${}^{a}$	B3LYP/6-31+G(d,p)	39.7	166.2	1.722
	PBE0/6-311++G(d,p)	40.3	168.5	1.747
	PBE0/6-311+G(d,p)	40.9	171.2	1.774
	PBE0/6-31++G(d,p)	39.8	166.6	1.727
	PBE0/6-31+G(d,p)	39.0	163.2	1.691
	M062x/6-311++G(d,p)	38.4	160.8	1.666
	M062x/6-311+G(d,p)	38.4	160.6	1.665
	M062x/6-31++G(d,p)	38.5	161.1	1.669
	M062x/6-31+G(d,p)	37.5	156.8	1.625
	M052x/6-311++G(d,p)	39.1	163.7	1.696
	M052x/6-311+G(d,p)	39.1	163.4	1.694
	M052x/6-31++G(d,p)	39.2	164.1	1.701
	M052x/6-31+G(d,p)	38.2	160.0	1.659
Methanol	B3LYP/6-311++G(d,p)	39.3	164.6	1.705
$\Delta H_{sol}\left(H^{+}\right)=-1070.8$ kJ/mol ${}^{b}$	B3LYP/6-311+G(d,p)	39.3	164.3	1.703
$\Delta H_{sol}\left(e^{-}\right)=-81.4$ kJ/mol ${}^{b}$	B3LYP/6-31++G(d,p)	39.0	163.0	1.690
$\Delta H_{sol}\left(H\right)=5$ kJ/mol ${}^{a}$	B3LYP/6-31+G(d,p)	38.1	159.3	1.651
	PBE0/6-311++G(d,p)	38.6	161.6	1.675
	PBE0/6-311+G(d,p)	39.3	164.3	1.703
	PBE0/6-31++G(d,p)	38.2	159.7	1.656
	PBE0/6-31+G(d,p)	37.3	156.3	1.619
	M062x/6-311++G(d,p)	36.8	153.9	1.595
	M062x/6-311+G(d,p)	36.7	153.7	1.593
	M062x/6-31++G(d,p)	36.8	154.2	1.598
	M062x/6-31+G(d,p)	35.8	149.9	1.553
	M052x/6-311++G(d,p)	37.5	156.8	1.625
	M052x/6-311+G(d,p)	37.4	156.5	1.622
	M052x/6-31++G(d,p)	37.6	157.2	1.629
	M052x/6-31+G(d,p)	36.6	153.1	1.587
Water	B3LYP/6-311++G(d,p)	39.4	164.9	1.709
$\Delta H_{sol}\left(H^{+}\right)=-1058.9$ kJ/mol ${}^{b}$	B3LYP/6-311+G(d,p)	39.3	164.6	1.706
$\Delta H_{sol}\left(e^{-}\right)=-102.0$ kJ/mol ${}^{b}$	B3LYP/6-31++G(d,p)	39.0	163.3	1.692
$\Delta H_{sol}\left(H\right)=-4$ kJ/mol ${}^{a}$	B3LYP/6-31+G(d,p)	38.2	159.6	1.655
	PBE0/6-311++G(d,p)	38.7	161.9	1.678
	PBE0/6-311+G(d,p)	39.3	164.6	1.706
	PBE0/6-31++G(d,p)	38.2	160.0	1.658
	PBE0/6-31+G(d,p)	37.4	156.6	1.623
	M062x/6-311++G(d,p)	36.9	154.2	1.598
	M062x/6-311+G(d,p)	36.8	154.0	1.596
	M062x/6-31++G(d,p)	36.9	154.5	1.601
	M062x/6-31+G(d,p)	35.9	150.1	1.556
	M052x/6-311++G(d,p)	37.5	157.1	1.628
	M052x/6-311+G(d,p)	37.5	156.8	1.626
	M052x/6-31++G(d,p)	37.6	157.5	1.632
	M052x/6-31+G(d,p)	36.7	153.4	1.590

^a^ Cf. Table 3, [54]. ^b^ Table 5, [55].

**Table 5:** Ionization enthalpies (IP${}_{H}$) of the H-atom in common environments (poorly) approximated as Kohn–Sham HOMO energy with reversed sign (Koopmans’ theorem). As already noted in the caption of Table 2, the results using the basis sets 6-311+G(d,p) and 6-311G(d,p), and 6-31+G(d,p) and 6-31G(d,p) are identical. Likewise, including more polarization functions, G(d,p) → G(2d,2p) or 6-311++(3df,3pd), has no impact on the values presented here.

Medium	Method	kcal/mol	kJ/mol	eV
Gas	B3LYP/6-311++G(d,p)	202.3	846.5	8.774
	B3LYP/6-311+G(d,p)	201.9	844.6	8.754
	B3LYP/6-31++G(d,p)	202.5	847.3	8.782
	B3LYP/6-31+G(d,p)	198.4	830.1	8.603
	PBE0/6-311++G(d,p)	210.5	880.8	9.128
	PBE0/6-311+G(d,p)	210.1	878.9	9.109
	PBE0/6-31++G(d,p)	210.6	881.0	9.131
	PBE0/6-31+G(d,p)	206.9	865.8	8.973
Benzene	B3LYP/6-311++G(d,p)	200.9	840.5	8.711
	B3LYP/6-311+G(d,p)	200.6	839.1	8.697
	B3LYP/6-31++G(d,p)	201.1	841.4	8.721
	B3LYP/6-31+G(d,p)	197.6	826.6	8.567
	PBE0/6-311++G(d,p)	209.1	874.8	9.067
	PBE0/6-311+G(d,p)	208.8	873.5	9.053
	PBE0/6-31++G(d,p)	209.2	875.3	9.071
	PBE0/6-31+G(d,p)	206.1	862.3	8.937
Toluene	B3LYP/6-311++G(d,p)	200.8	840.3	8.709
	B3LYP/6-311+G(d,p)	200.5	839.0	8.695
	B3LYP/6-31++G(d,p)	201.2	841.2	8.719
	B3LYP/6-31+G(d,p)	197.5	826.5	8.566
	PBE0/6-311++G(d,p)	209.0	874.6	9.064
	PBE0/6-311+G(d,p)	208.7	873.3	9.051
	PBE0/6-31++G(d,p)	209.1	875.1	9.070
	PBE0/6-31+G(d,p)	206.1	862.1	8.935
Ethanol	B3LYP/6-311++G(d,p)	199.9	836.2	8.667
	B3LYP/6-311+G(d,p)	199.6	835.3	8.657
	B3LYP/6-31++G(d,p)	200.1	837.2	8.677
	B3LYP/6-31+G(d,p)	197.0	824.0	8.541
	PBE0/6-311++G(d,p)	208.1	870.5	9.022
	PBE0/6-311+G(d,p)	207.9	869.6	9.013
	PBE0/6-31++G(d,p)	205.5	859.7	8.910
	PBE0/6-31+G(d,p)	208.2	871.1	9.029
Methanol	B3LYP/6-311++G(d,p)	199.8	836.1	8.665
	B3LYP/6-311+G(d,p)	199.6	835.2	8.656
	B3LYP/6-31++G(d,p)	200.1	837.1	8.676
	B3LYP/6-31+G(d,p)	196.9	824.0	8.540
	PBE0/6-311++G(d,p)	208.0	870.4	9.021
	PBE0/6-311+G(d,p)	207.8	869.6	9.012
	PBE0/6-31++G(d,p)	208.2	871.0	9.028
	PBE0/6-31+G(d,p)	205.5	859.7	8.910
Water	B3LYP/6-311++G(d,p)	199.8	835.9	8.663
	B3LYP/6-311+G(d,p)	199.6	835.0	8.654
	B3LYP/6-31++G(d,p)	200.0	837.0	8.674
	B3LYP/6-31+G(d,p)	196.9	823.9	8.539
	PBE0/6-311++G(d,p)	208.0	870.2	9.019
	PBE0/6-311+G(d,p)	207.8	869.4	9.011
	PBE0/6-31++G(d,p)	208.1	870.9	9.026
	PBE0/6-31+G(d,p)	205.4	859.5	8.908

**Table 6:** Ionization enthalpies (IP${}_{H}$) of the H-atom in common environments (poorly) approximated as Kohn–Sham HOMO energy with reversed sign (Koopmans’ theorem). Results using Minnesota functionals M062x and M052x and basis sets 6-31+G(d,p).

Medium	Method	kcal/mol	kJ/mol	eV
Gas	M062x	233.4	976.7	10.122
Benzene		232.6	973.2	10.086
Toluene		232.6	973.1	10.085
Ethanol		232.0	970.7	10.060
Methanol		232.0	970.6	10.060
Water		232.0	970.5	10.058
Gas	M062x	237.1	992.1	10.282
Benzene		236.3	988.6	10.246
Toluene		236.3	988.5	10.245
Ethanol		235.7	986.1	10.221
Methanol		235.7	986.1	10.220
Water		235.7	986.0	10.220

**Table 7:** The enthalpies of reaction (in kcal/mol) of peonidin according to [24].

Enthalpy	Gas	Water
BDE	81.50	78.92
IP	235.37	138.36
PDE	159.88	254.51
PA	−120.49	−88.96
ETE	515.74	483.38
IP+PDE	395.25	392.87
PA+ETE	395.25	394.42
$IP+PDE-BDE(\overset{?}{=}{IP}_{H})$	313.75 (okay)	313.95 (wrong)
$PA+ETE-BDE(\overset{?}{=}{IP}_{H})$	313.75 (okay)	315.50 (wrong)
${IP}_{H}$	314.7 ${}^{a}$	32.8 ${}^{b}$

^a^ Cf. Table 3. ^b^ Table 4.

**Table 8:** The enthalpies of reaction (in kcal/mol) characterizing the antioxidant activity of atorvastatin (ATV) and its ortho- and para-hydroxy metabolites (o-ATV, p-ATV) in methanolic phase [22] (and corrections reprinted [22]) satisfy the two theorems stated in this paper. The values of the difference between IP+PDE = PA+ETE and BDE, identical for all molecular species and positions of H-atom abstraction, are equal to the ionization of the H-atom in methanol computed at the same B3LYP/6-31+G(d,p) level of theory (cf. Table 4).

Molecule	Position	BDE	IP	PDE	SETPT	PA	ETE	SPLET	IP${}_{H}$
ATV	1-OH	91.4	107.0	22.4	129.4	23.8	105.7	129.4	38.1
	2-OH	104.2		35.3	142.3	46.7	95.6	142.3	38.1
	3-OH	105.2		36.3	143.2	61.5	81.8	143.2	38.1
	4-NH	90.2		21.3	128.3	44.4	83.9	128.3	38.1
o-ATV	1-OH	91.2	106.9	22.4	129.3	23.8	105.5	129.3	38.1
	2-OH	104.2		35.4	142.3	46.8	95.5	142.3	38.1
	3-OH	105.1		36.3	143.2	61.5	81.7	143.2	38.1
	4-NH	89.3		20.5	127.4	49.0	78.4	127.4	38.1
	5-OH	77.5		8.7	115.6	34.4	81.2	115.6	38.1
p-ATV	1-OH	90.7	106.2	22.6	128.8	23.8	105.0	128.8	38.1
	2-OH	104.2		36.0	142.2	46.8	95.4	142.2	38.1
	3-OH	105.1		37.0	143.2	58.2	85.0	143.2	38.1
	4-NH	85.5		17.4	123.6	43.8	79.0	123.6	38.1
	5-OH	77.4		9.2	115.4	37.9	77.5	115.4	38.1

**Table 9:** The enthalpies of reaction (in kJ/mol) that quantify the antioxidant activity of vitamin B3 computed for gaseous and aqueous phases using the methods indicated. Notice that there is no difference between unrestricted (UB3LYP) and restricted open-shell (ROB3LYP) methods in calculating the PA values, and for this reason the the latter are written in parentheses. Values claimed to be for the aqueous phase of [23] are also included, but some thereof seem rather to be for gas.

Method	Basis Set	Phase	BDE	IP	PDE	PA	ETE
UB3LYP	6-311G(d,p)	gas	437.1	879.9	878.7	1432.5	326.1
ROB3LYP	6-311G(d,p)	gas	443.6	884.3	880.8	(1432.5)	332.6
UB3LYP	6-311++G(d,p)	gas	438.6	891.8	868.6	1397.4	363.0
ROB3LYP	6-311++G(d,p)	gas	445.3	896.1	870.9	(1397.4)	369.6
UB3LYP	6-311G(d,p)	aqueous	436.3	584.2	18.6	154.2	448.6
ROB3LYP	6-311G(d,p)	aqueous	442.7	588.5	20.7	(154.2)	455.1
UB3LYP	6-311++G(d,p)	aqueous	437.2	595.1	8.9	117.8	486.2
ROB3LYP	6-311++G(d,p)	aqueous	443.9	599.5	11.2	(117.8)	492.9
B3LYP ${}^{a}$	6-311G**	aqueous (claimed)	441	892	869	149	478

^a^ From [23].

**Table 10:** Combined enthalpies of reaction (in kJ/mol) for the three antioxidant mechanisms (HAT, SET-PT, and SPLET) for vitamin B3 in the gas and aqueous phase along with those extracted from [23]. Notice that our estimates satisfy both Theorems 1 and 2 while those of [23] do not.

Method	Basis Set	Phase	BDE + PDE	PA + ETE	IP + PDE − BDE	PA + ETE − BDE	IP${}_{H}$
UB3LYP	6-311G(d,p)	gas	1758.6	1758.6	1321.5	1321.5	1321.5
ROB3LYP	6-311G(d,p)	gas	1765.1	1765.1	1321.5	1321.5	1321.5
UB3LYP	6-311++G(d,p)	gas	1760.4	1760.4	1321.8	1321.8	1321.8
ROB3LYP	6-311++G(d,p)	gas	1767.1	1767.1	1321.8	1321.8	1321.8
UB3LYP	6-311G(d,p)	aqueous	602.8	602.8	166.5	166.5	166.5
ROB3LYP	6-311G(d,p)	aqueous	609.2	609.2	166.5	166.5	166.5
UB3LYP	6-311++G(d,p)	aqueous	604.0	604.0	166.8	166.8	166.8
ROB3LYP	6-311++G(d,p)	aqueous	610.7	610.7	166.8	166.8	166.8
B3LYP ${}^{a}$	6-311G**	aqueous (claimed)	1761	627	1320	186	166.5

^a^ From [23].

## Data Availability

The data that support the findings of this study are available from the author upon reasonable request.
